# *HRness* in Breast and Ovarian Cancers

**DOI:** 10.3390/ijms21113850

**Published:** 2020-05-28

**Authors:** Elizabeth Santana dos Santos, François Lallemand, Ambre Petitalot, Sandrine M. Caputo, Etienne Rouleau

**Affiliations:** 1 Department of Medical Biology and Pathology, Gustave Roussy, Cancer Genetics Laboratory, Gustave Roussy, 94800 Villejuif, France; elizabeth.santanadossantos@gmail.com; 2 Department of Clinical Oncology, A.C. Camargo Cancer Center, São Paulo 01509-010, Brazil; 3 Department of Genetics, Institut Curie, 75005 Paris, France; francois.lallemand@curie.fr (F.L.); ambre.petitalot@curie.fr (A.P.); sandrine.caputo@curie.fr (S.M.C.); 4 PSL Research University, 75005 Paris, France

**Keywords:** homologous recombination deficiency, DNA repair, breast cancer tumorigenesis, ovarian cancer tumorigenesis, BRCA1, BRCA2, hereditary breast cancer, hereditary ovarian cancer

## Abstract

Ovarian and breast cancers are currently defined by the main pathways involved in the tumorigenesis. The majority are carcinomas, originating from epithelial cells that are in constant division and subjected to cyclical variations of the estrogen stimulus during the female hormonal cycle, therefore being vulnerable to DNA damage. A portion of breast and ovarian carcinomas arises in the context of DNA repair defects, in which genetic instability is the backdrop for cancer initiation and progression. For these tumors, DNA repair deficiency is now increasingly recognized as a target for therapeutics. In hereditary breast/ovarian cancers (HBOC), tumors with *BRCA1/2* mutations present an impairment of DNA repair by homologous recombination (HR). For many years, *BRCA1/2* mutations were only screened on germline DNA, but now they are also searched at the tumor level to personalize treatment. The reason of the inactivation of this pathway remains uncertain for most cases, even in the presence of a HR-deficient signature. Evidence indicates that identifying the mechanism of HR inactivation should improve both genetic counseling and therapeutic response, since they can be useful as new biomarkers of response.

## 1. Tumorigenesis of Breast and Ovarian Epithelial Carcinomas

Breast carcinomas arise in the terminal duct lobular units of the collecting ducts (the functional unit of the breast), as a consequence of a continuum of lesions and molecular alterations from normal glands to malignant tumors. The two main localizations of invasive cancers are inside the milk duct for ductal carcinomas and inside the milk glands (lobules) for lobular carcinomas.

The cell origin and the molecular alterations that drive breast carcinogenesis differ among the subtypes. The work of Perou and Sorlie allowed the subdivision of breast cancers in five subtypes, distinguished by differences in their gene expression patterns with distinct clinical behaviors: basal, erb-b2 receptor tyrosine kinase 2 (ERBB/HER2), luminal A, luminal B, and normal breast-like [1,2].

Evidence suggests that at the molecular level, these different molecular subtypes evolve along two different pathways of progression: (1) low-grade-like pathway, characterized by gain of 1q, loss of 16q, infrequent amplification of 17q12, gene expression signature associated with estrogen receptor (ER) phenotype, diploid or near-diploid karyotypes, and low tumor grade, including luminal A and part of luminal B tumors, and (2) the high-grade-like pathway, characterized by loss of 13q, gain of chromosomal region 11q13, amplification of 17q12 (region of *ERBB2*/*HER2* gene), and expression of signature genes involved in the cell cycle and cellular proliferation, including intermediate-high grade tumors such as HER2-positive and triple-negative breast cancer (TNBC or breast cancer negative for ER, progesterone receptor (PR), and *ERBB2*/*HER2* gene amplification) [3]. For breast cancer, the inactivation pathway can be based either on the inactivation of *TP53*, or the inactivation of *PTEN* [4]. Somatic mutations in *TP53* are present in 20–40% of breast cancers [5,6], whereas mutations in *PTEN* are less frequent (1–5% of breast cancers). Studies have shown that *TP53* mutations can occur in ductal carcinoma in situ (DCIS) before the development of invasive breast cancer, which implies an important role for TP53 inactivation early in breast cancer [7]. While these mutations are almost null in low-grade DCIS, they are reported at a frequency of 30–40% in high-grade DCIS. TP53 and PTEN alterations mainly occur in ER-negative cancers, whereas ER-positive have more *GATA3, PIK3CA*, and *CCND1* alterations [8].

Ovarian carcinomas correspond to 90% of ovarian cancers and comprise different subtypes of disease with specific morphologies and molecular patterns. It has been hypothesized that high-grade serous ovarian carcinomas (HGSOC) originate from pre-malignant lesions in the tubas (serous tubal intraepithelial carcinoma) instead of the ovary itself, since both share the same morphological and molecular features, which involves mutations in the *TP53* gene as an early event [9,10]. Atypical lesions within the fimbriated end of the fallopian tube (serous tubal intraepithelial carcinomas (STIC)) display similar morphology and *TP53* signatures as HGSOC, suggesting that the neoplastic process may originate at these tubal lesions and shed into the ovary, where they aggressively progress [11]. Compelling data suggests the same origin for low-grade serous carcinomas, but that they progress from benign serous cystadenoma to borderline serous tumors and then on to low-grade carcinomas.

Integrated genomic analysis led to the shift that ovarian cancer was not just one disease, but rather several distinct diseases presenting different histological and molecular features. HGSOC are characterized by nearly universal *TP53* abnormalities, also detected in endometrioid and other high-grade diseases. This subtype also presents high genomic instability, somatic DNA copy-number changes, and whole genome duplications. Homologous recombination deficiency (HRD) is present in about 50% of HGSOC. Overall, *TP53* mutations occur in 96% of cases, *BRCA1* and *BRCA2* mutations in 22% of cases (15–20% of these are germline), and additional somatic mutations in six other genes are identified in 2–6% of cases (*NF1, RB1, CDK12, FAT3, CSMD3*, and *GABRA6).* Recent molecular analysis, which was based on the profile of RNA and microRNA expression, stratified HGSOC into four different prognostic subtypes (C1-mesenchymal, C2-immune, C3-differentiated, C4-proliferative) and seven copy-number signatures. However, contrary to breast cancer, the molecular stratification is not yet validated for accurate prediction of drug sensitivity and/or resistance to treatment [12,13,14,15,16,17].

Breast and ovarian cancers are mostly carcinomas, originated from epithelial cells that are in constant division and subjected to cyclical variations of the estrogen stimulus during the female hormonal cycle, therefore being vulnerable to DNA damage. A portion of breast and ovarian carcinomas arises in the context of DNA repair defects, in which genetic instability is the backdrop for cancer initiation and progression. This is particularly relevant for triple-negative breast cancers and high-grade serous ovarian cancers, for which DNA repair deficiency is increasingly recognized as a target for therapeutics.

### 1.1. Maintenance of Genome Integrity

#### 1.1.1. Cell Cycle, DNA Repair, and Apoptosis

The cell cycle is divided into 4 phases: G1 (preparation of the DNA replication), S (DNA replication), G2 (preparation of the mitosis), and M (Mitosis). When a cell is out of the cell cycle, it is in the G0 phase. The cell cycle is controlled by different cyclin-dependent kinases (CDKs). Each CDK is specifically linked to a cyclin which is crucial for its kinase activity. The different CDK-cyclin dimers modulate the progression of cells through the cell cycle and each CDK-cyclin complex is specific of one or several phase(s) of the cell cycle. The CDK2-cycline E dimer modulates the G1/S transition, and the dimer CDK1-cycline A modulates the G2 and S phases. The CDK1-cycline B dimer modulates the M phase [18]. During the G1 phase, CDK4-cyclin D and CDK6-cyclin D phosphorylate the protein RB. This phosphorylation inhibits the RB-E2F association. Once liberated from RB, the transcriptional activator E2F activates the transcription of genes indispensable for the DNA replication or S phase [19]. The transition to each phase of the cell cycle is controlled by proteins such as CDK12 [18]. The regulation of CDK-cyclin complexes are mainly assured through phosphorylation and dephosphorylation cycles [20]. During S phase, the quantity of DNA is doubled with the replication forks. The double-stranded DNA is separated into single-stranded DNA, allowing the recruitment of replication protein A (RPA) and then the loading of the replicative DNA polymerases and proliferating cell nuclear antigen (PCNA) sliding clamps [21]. Three DNA polymerases—Pol α, Pol δ, and Pol ɛ—are essential for DNA replication. After Pol α initiates DNA synthesis, Pol δ takes over on the lagging and Pol ɛ takes over on the leading strand, performing the bulk of replication with very high fidelity [22]. The main actors are the polymerases Pol ε (coded by the *POLE* gene) and Pol δ (coded by the *POLD1* gene). Dysfunction of these proteins generates intrinsic DNA errors [23].

**Figure 1:**
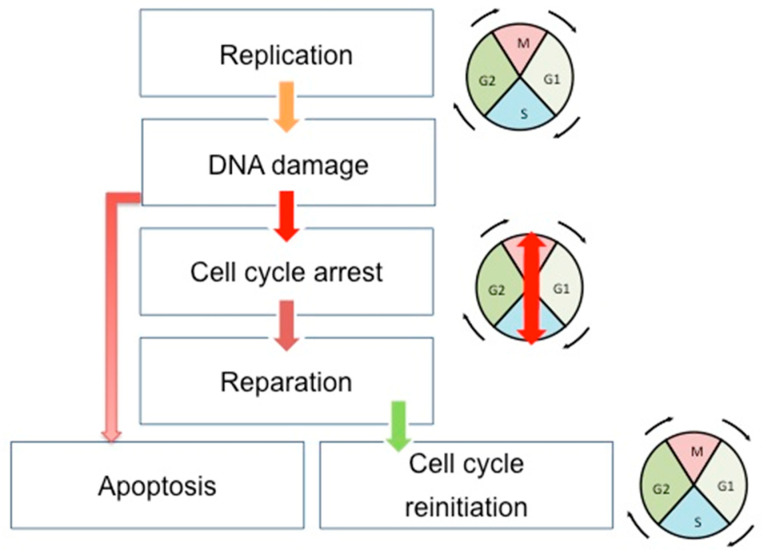
Schematic view of the correlation between cell cycle, DNA repair, and apoptosis pathways. During the cell cycle, if the DNA error is correctly repaired, the cells can restart their progression through the cell cycle. However, if the DNA damages are too important, the cells undergo apoptosis. Double-headed arrows indicate the evolution of the cell cycle phases: M (Mitosis), G1, S (Synthesis), and G2.

Cell cycle, DNA repair, and apoptosis are tightly connected to maintaining the genome integrity of cells and tissues in development and in constant division (Figure 1). The cell cycle, once initiated, could face DNA damage. If so, cells stop their progression through the cell cycle at specific points (cell cycle checkpoints) to correctly repair the errors in the DNA. This is achieved through the interaction existing between the CDKs and the proteins sensitive to DNA alterations. After the DNA repair, the cells can restart their progression through the cell cycle. However, if the DNA damages are too important, the cells undergo apoptosis. There are three cell cycle checkpoints: G1/S, S, and G2/M checkpoints [24]. Moreover, three canonical S-phase "checkpoint pathways” are involved in the maintenance of DNA integrity: the replication checkpoint detects the stalled replication fork, the S-M checkpoint blocks mitosis until the entire genome has been successfully duplicated, and the intra-S phase checkpoint, whose main regulators are ATR and ATM kinases, is sensitive to double-strand-breaks (DSBs) [25]. This system will detect DNA errors, leading to the stop of the cell cycle and the direct reparation of these errors.

#### 1.1.2. Reparation Mechanisms of DNA

**Figure 2:**
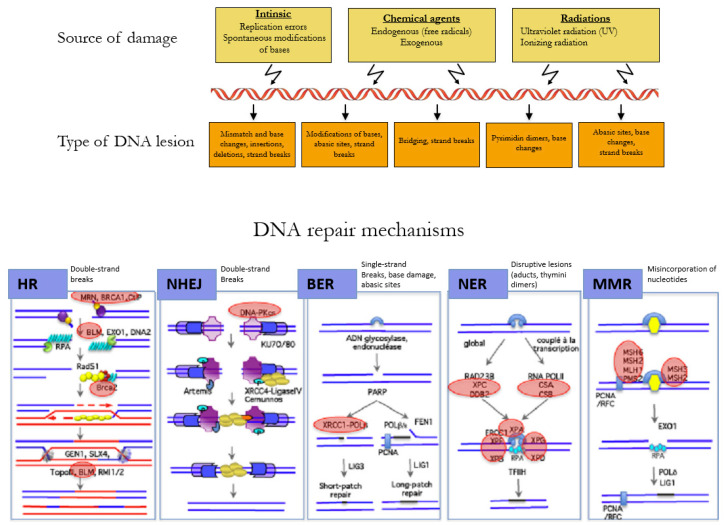
Mechanisms of DNA repair according to the type of lesion. DNA double-strand breaks are repaired by the error-free pathway Homologous Recombination (HR) or the error-prone pathway Non-Homologous End Joining (NHEJ). Single-strand breaks are repaired by base excision repair (BER), bulkier single-strand lesions that distort the DNA helical structure are repaired by Nucleotide Excision Repair (NER), and misincorporation of nucleotides resulting in mismatches in the DNA sequence are repaired by Mismatch Repair (MMR). Adapted with permission from Rass et al. [30].

The DNA of a cell can suffer between 10,000 to 100,000 damages a day caused by environmental agents and endogenous processes, such as transcription and replication of the DNA (Figure 2) [26]. The choice of the repair mechanism used by the cell to repair its DNA is determined by the type of lesion and the position of the cell in the cell cycle. Single-strand breaks are repaired by base excision repair (BER) [27], bulkier single-strand lesions that distort the DNA helical structure are repaired by Nucleotide Excision Repair (NER) [28], and misincorporation of nucleotides resulting in mismatches in the DNA sequence are repaired by Mismatch Repair (MMR) [29].

**Figure 3:**
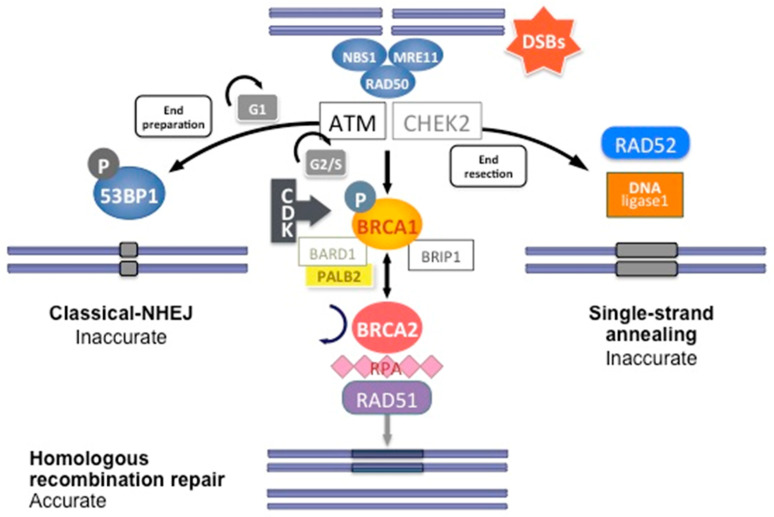
DNA double-strand break repair by homologous recombination. Coordinated interaction of BRCA1 and BRCA2 with other HR proteins is required to repair double-strand breaks.

DNA double-strand breaks (DSBs) are carried out by two major mechanisms that differ in their fidelity and template requirement: Non-Homologous End Joining (NHEJ) and Homologous Recombination (HR). NHEJ is an error-prone pathway that does not use a template for DNA repair but simply ligates the broken DNA ends together, which leads to an accumulation of errors such as little insertions or deletions [31]. However, HR repair is a highly conserved mechanism that enables the accurate repair of DNA double-strand breaks by using the intact sister chromatid as a template for repair, thereby maintaining the sequence integrity (Figure 3). As it requires a template for repair, it occurs mainly during the late S and G2 phases of the cell cycle. BRCA1 and BRCA2 are key components of this pathway that involves the coordinated interaction of BRCA1 and BRCA2 with other DNA repair proteins such as ATM, CHEK2, BARD1, BRIP1/BACH1, MRE11, RAD50, NBN/NBS1, RAD51C, RAD51D, and PALB2. In cells that are deficient of BRCA1⁄2, the repair of DNA double-strand breaks relies on the error-prone NHEJ pathway [32].

The reparation mechanisms of NHEJ and HR are not involved equally during the cell cycle. In G0 and M phases, CDK activity is low and no sister chromatid is available, favoring NHEJ pathways. NHEJ is mainly present during the G1 phase, whereas HR is mainly present during the S phase. In the M phase, both HR and NHEJ repair are extremely reduced, and DSB that arise during mitosis are repaired by single-strand annealing (a DNA damage repair mechanism that uses homologous repeats to bridge DSB ends, causing a deletion rearrangement between the repeats), resulting in large-scale chromosomal rearrangements. CDK activity, which increases in the S and G2 phases of the cell cycle, also favors BRCA1 activation and DNA repair by HR.

In short, HR DNA repair begins after recognition of the 5′ends of the double-strand DNA break by the MRN complex (**M**RE11-**R**AD50-**N**BS1) (Figure 3). This complex recruits Ataxia telangiectasia mutated (ATM), a protein kinase. ATM subsequently phosphorylates downstream proteins, particularly BRCA1, as well as CHEK1 and CHEK2, which are two serine/threonine-protein kinases inducing cell cycle arrest at the G1/S and G2/M cell cycle checkpoints, allowing DNA damages repair [24]. The phosphorylation of BRCA1 by ATM induces its recruitment to DNA damage sites and its binding to the BRCA1-associated RING domain (BARD1), a E3 ubiquitin-protein ligase essential for BRCA1 stability [24]. CDK activity, which increases in the S and G2 phases of the cell cycle, also favors BRCA1 activation and DNA repair by HR. BRCA1 activation then allows extensive 5′ end resection to produce 3′ single-stranded DNA and the induction of the RAD51 loading to the single-stranded DNA by the BRCA1/BRCA2/PALB2 complex. PALB2 recruits BRCA2 and RAD51 to DNA break sites by enhancing BRCA1–BRCA2 interaction and binding DNA with high affinity for D loop [24]. DNA is then repaired using the homologous region of the chromatid as a replicative template.

**Figure 4:**
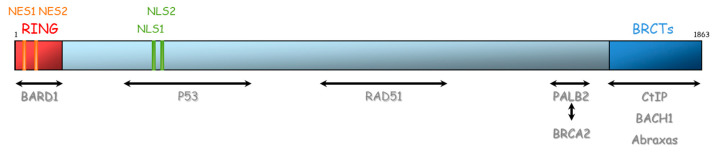
Representation of BRCA1 protein and its domains of interaction with other proteins. NES: Nuclear export signal, NLS: nuclear localization signal, BRCT: BRCA1 carboxyl-terminal, BARD1: BRCA1-associated RING domain.

BRCA1 and BRCA2 are therefore essential proteins involved in HR. Their dysfunction leads to genomic instability, which is a hallmark of cancer [33]. The *BRCA1* gene is located on chromosome 17 (17q21) and encompasses 24 exons. It was originally mapped in 1990 and subsequently cloned in 1994 [34]. This gene encodes a 1863-amino acid protein that contains at the N-terminus, a nuclear export signal (NES), and a zinc-binding RING domain (Exons 2–7, aa 8–96, Figure 4) [35,36]. The RING domain heterodimerizes with BARD1 to form an E3 ubiquitin ligase. The main function of BRCA1⁄BARD1 complex is its E3 ubiquitin ligase activity (post damage) at double-strand break sites, which results in the ubiquitination of other proteins involved in DNA damage repair, such as CtIP and H2AX [37,38,39]. Ubiquitinated CtIP binds to chromatin to manage G2⁄M checkpoint control. Ubiquitinated H2AX remodels chromatin so that it becomes accessible for DNA repair machinery. In its carboxyl-terminus, there are tandem repeats of two BRCA1 carboxyl-terminal (BRCT) domains (Exons 16–24, aa 1646–1855). Each comprises of about 100 amino acids and engages in forming functional macromolecules complexes with partner proteins (CtIP, BACH1, Abraxas). More central, BRCA1 has two nuclear localization signals (NLS): one DNA binding domain and one Serine-glutamine (SQ) cluster domain containing several threonine and serine residues which can become phosphorylated [40]. BRCA1 also interacts with BRCA2 via the bridging protein PALB2 (partner and localizer of BRCA2) through BRCA1 coiled-coil domain during RAD51 recruitment to double-strand breaks [41].

**Figure 5:**
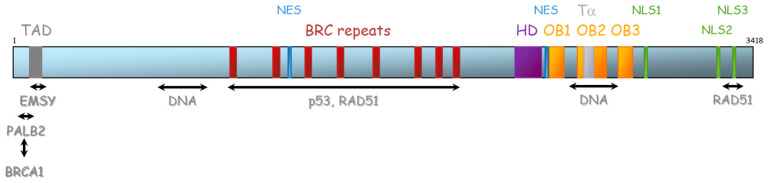
Representation of BRCA2 protein and its domains of interaction with protein partners. TAD: transactivation domain, NES: nuclear export domain, HD: helical domain, OB: oligosaccharide binding, Tα: Tower alpha, NLS: Nuclear localization signal.

The *BRCA2* gene is located on chromosome 13 (13q12.3) and encompasses 27 exons (26 coding exons). *BRCA2* encodes a 3418-amino acid protein that also contains motifs that mediate its interaction with partner proteins (Figure 5). Currently, three BRCA2 regions have been described as particularly important for HR function: (1) N-terminal PALB2-binding site (aa 10–40), that physically links BRCA1 and BRCA2 and is critical for maintenance of the DNA repair function of these proteins [41,42,43,44]. The same region has also been implicated in binding to EMSY [45]. (2) BRC repeat (aa 900–2000) corresponds to eight consecutive motifs of about 35 residues located in the central region of the protein (in the exon 11), with a well-described function of interaction with RAD51 and other partners [46,47,48], and (3) C-terminal region (aa 2459–3190), composed of three oligosaccharide binding folds (or OB folds), a helical domain, and a tower domain that together constitute the DNA binding region [49,50,51]. Another RAD51 binding region (aa 3265–3330) was shown in the C-terminal region [52]. Recently, Carreira et al. showed a new DNA interaction with the N-terminal [50] and Shibuya et al. showed an interaction with MEILB2/HSF2BP with aa 2117–2339 [53].

In addition to the maintenance of genomic integrity, BRCA1 and BRCA2 have other cellular functions whose failure might also be related to carcinogenesis. BRCA1 is involved in checkpoint regulation during cell cycle, which is a strategy that transiently inhibits DNA synthesis allowing for the repair of DNA lesions [54]. BRCA1 participates in the maintenance of centrosome number during late S and G2⁄M phases of the cell cycle and in the regulation of apoptosis [55]. BRCA1 regulates its expression at the RNA transcription level and through participation on chromatin remodeling [56,57]. Contrary to BRCA1, the role of BRCA2 in transcriptional and cell cycle regulation is less certain but some studies support such roles [58]. BRCA2 has been shown to play a role in a number of mitotic processes, including the spindle assembly checkpoint, cytokinesis, and daughter cell abscission [53,59].

#### 1.1.3. Dysfunction of the Repair Pathways

(1) Protein Expression Alteration and Mutation in the Coding Regions

Most of the genes implicated in DNA repair have been classified as tumor suppressor genes. Their dysfunction can be related to the absence of the protein or to the inactivation of functional domains. The main cause of dysfunction is the absence of protein due to mutations in the coding region. Mutations in genes linked to HR (HR genes) have been reported in breast and ovarian cancers [15,60,61] and in recent years, several studies have evaluated the consequences of the absence of BRCA1/2 expressions. However, presently, there is no appropriate antibody to routinely test the absence of the BRCA1 or BRCA2 protein in tumors presenting *BRCA1/2* mutations by immunohistochemistry. Despite the lack of such antibody, some studies showed a correlation between *BRCA1* mutation status and protein expression for ovarian carcinomas [62,63]. For BRCA2, data seems to be more heterogeneous and difficult to interpret [64]. No correlation was found for *BRCA2* expression in prostate cancer [65]. However, there are a few examples of interest for performing immunohistochemistry to evaluate BRCA1 protein [62] and nuclear expression for BRCA2-associated tumor [66]. The functional RAD51 foci assay (which showed to be highly discriminative of HRD [67]) could emerge to identify any HR deficiency.

(2) Presence of Missense Variants

A genetic variant can be defined as an alteration in the most common DNA nucleotide sequence (of reference sequence). They can be inferred as pathogenic since they result in predicted truncating or null proteins, and/or are frequent enough in breast–ovarian cancer families that their risk of disease can be estimated directly [68].

The presence of missense variant in functional domains can hurdle the pathway of reparation. Pathogenic missense variants generally have impact on domains directly implied in the DNA reparation activity, such as the BRCT and RING domains of the *BRCA1* gene.

(3) Promoter Methylation and miRNA

The deregulation of the protein expression can be related to level of expression without any mutation in the coding sequence. In addition to mutations in *BRCA1⁄2* genes, the presence of genetic instability may be a consequence of mutations or epigenetic silencing of *BRCA1⁄2* or other HR genes. The main possibility is related to promoter methylation. Aberrant *BRCA1* promoter methylation is seen in 5%–30% of ovarian cancers [69,70] and in 11%–14% of sporadic breast cancer. It is more frequent (~30%) among TNBC [69,71]. Promoter hypermethylation in ovarian and TNBC samples have also been described in other HR genes, such as *PALB2*, *ATM*, *RAD50*, *RAD51C*, and *FANCF* [72,73]. In contrast, *BRCA2* promoter hypermethylation is a very rare event and certainly not well defined in breast and ovarian tumorigenesis. Additionally, BRCA post-transcriptional downregulation through miRNA has been described in breast and ovarian carcinogenesis, which could also explain cases sharing BRCA histopathological features with no mutation identified [74].

(4) Transcription and Post-Translational Regulation

The regulation of the transcription of certain genes involved in DNA repair has been well described. The role of CDK12 has been described as an activator of the HR genes promoters [75]. The protein EMSY is more inclined to have a negative impact on the expression, which explains the role of the amplification of its gene [76]. Moreover, steroid hormones may affect *BRCA1* expression indirectly, by altering the proliferative status of the cell rather than acting directly on DNA sequences. So far, no estrogen receptor (ER) site has been identified on *BRCA1* regulatory regions [77].

Another level of alteration could happen in the post-translational regulation. Some post-translational alterations, such as phosphorylation, can be clearly responsible for activation or inactivation of a pathway [78]. The mechanism implying ubiquitin modification is also known to limit the activity of some proteins by accelerating the intracytoplasmic destruction of them. This mechanism has been described with HR proteins [79].

#### 1.1.4. Compensatory Mechanisms to Other Reparation Pathways

Since there are several DNA repair pathways, some compensatory mechanisms have been observed [80]. For example, if the HR pathway is inactivated, the BER and the alternative NHEJ DNA repair will try to compensate for it imperfectly [30]. Moreover, since HR requires a full coordination of different proteins, the impact on the pathway will probably differ according to the protein affected.

### 1.2. Signature

The inefficiency of the DNA repair mechanism generates a scenario of genetic instability. Mutational signatures are then designed to identify the homologous recombination-deficient (HRD) phenotype and characterize a larger population which can benefit from DNA damaging agents, extending beyond *BRCA* mutant tumors. Every signature serves as an imprint of a distinct DNA damage and repair process operative in the tumor at some point during tumoral development.

This includes signatures based on the evaluation of the following: (1) Copy number alteration [81] (CNA) profiles, which are determined by the identification of DNA gains or losses in the tumor. CNA can be evaluated by comparative genomic hybridization array (aCGH), multiplex ligation-dependent probe amplification (MLPA), or single-nucleotide polymorphism (SNP) arrays. (2) Loss of heterozygosity (LOH) score, evaluated by the imbalance in the ratio of parental alleles from the normal 1:1 [82]. (3) Telomeric allelic imbalance, which calculates the allelic imbalance extending from the site of DNA damage to the telomere [83,84]. (4) Large-scale transitions that consist of chromosome breaks (translocations, inversions, or deletions) of at least 10 Mb between adjacent regions [85], and (5) mRNA and miRNA expression [86]. (6) Mutational signature based on the experience of the TCGA (The Cancer Genome Atlas Program)—signature 3 [87]. Finally, two scores were developed combining different methods to improve the sensitivity to identify the BRCAness phenotype: myChoice HRD test (Myriad Genetics) combines measures of LOH, TAI, and LST [88], and Foundation Medicine HR score combines measures of *BRCA1⁄2* mutation status and percentage of LOH [89]. The threshold of composite scores have been described and validated in prospective clinical trials [90].

HRD tumors represent up to 50% of HGSOC and more than 20% of basal breast cancers, but a *BRCA* mutation is identified in only 20% of them [14,91]. According to the results of recent trials, even if the tumor is sporadic, the identification of an HRD phenotype helps in personalizing therapy. The comprehension of breast- and ovarian-associated carcinogenesis has evolved from solely mutation identification in candidate genes onwards to the integration of a large volume of genomics and transcriptome data, revealing recurrently altered pathways and signatures of mutational processes. All methods described above (individually or in combination) were able to discriminate HR-deficient tumors which were correlated with responsiveness to platinum and PARPi (Poly (ADP-ribose) polymerase (PARP) inhibitors), and resulted in improved outcomes.

### 1.3. Temporal Order and Somatic Tumor-Driving Events of BRCA-Associated Tumorigenesis

In relation to tumorigenesis specifically related to *BRCA1* and *BRCA2,* data suggest that *BRCA* bi-allelic inactivation renders the cell vulnerable to genomic instability, being the background for successive mutations that culminate in cancer development. Conforming to this theory, Von Waldhe et al. recently demonstrated concordance between HRD scores across different regions of the same BRCA-associated breast cancer, indicating that HRD affects the entire primary tumor and corresponds to a founding event [92].

As typical tumor suppressor genes, the inactivation of the second allele of *BRCA1⁄2* is presumed to be a rate-limiting step [93]. LOH is the most common second hit event of breast and ovarian *BRCA1⁄2*-associated carcinogenesis. It is a consequence of large deletions, genomic rearrangements, incorrect mitosis, or deficient DNA repair. It has been reported in 90% (breast) and 91% (ovarian) of *BRCA1*-associated cancers and in 54% (breast) and 84% (ovarian) of *BRCA2*-associated cancers [94]. Alternative second-hit mechanisms, such as somatic inactivating point mutations, have been described in a small minority of *BRCA1*-associated breast and ovarian cancers [95,96]. Furthermore, hypermethylation of the *BRCA1* promoter has also been responsible for the silencing of the wild-type allele, but only in a minority of the cases [97,98].

Van Heetvelde et al. described the panorama of second-hit events in breast and ovarian cancers from patients harboring germline *BRCA1⁄2* mutations. Copy neutral LOH was the most prevalent mechanism of wild-type (WT) allele inactivation (detected in 69% of breast cancers and 67% in ovarian cancers). However, most intriguing was that only a minority of tumors (35% breast and 47% ovarian cancers) presented loss of the WT allele in all cancerous cells but in the majority of the cases, different mechanisms of WT allele inactivation were present in the same tumor [99]. Moreover, somatic intragenic deletions and methylated subclones were found in combination with partial LOH.

It has been suggested that heterozygous mutations affecting *BRCA1* and *BRCA2* might be enough for carcinogenesis, even when the remaining WT allele remains expressed. Recent genomic studies have showed that a significant fraction of cancers arising in *BRCA* mutation carriers retain a functional WT allele. Jonsson et al. observed this in 25% of breast and ovarian cancers with *BRCA1⁄2* mutations [100]. In line with this, Maxwell et al. observed the retention of the WT allele in 46% breast and 16% ovarian *BRCA2*-associated cancers. However, it was less frequent for *BRCA1* breast (7%) and ovarian (10%) cancers [94]. The prevalence of 8% was globally estimated in a large panorama of tumors and clearly more frequent in lung cancer (up to 20%) [100]. Furthermore, some lines of evidence suggest that the presence of heterozygous truncating *BRCA1* and *BRCA2* mutations may render cells vulnerable to haploinsufficiency, when exposed to replication stress [101].

### 1.4. Conclusion for Routine Practice

Currently, the molecular testing with the highest level of evidence is based on the screening of the coding sequences of *BRCA1* and *BRCA2* genes as the main drivers of HR pathway inactivation. This analysis is most often performed through NGS (Next-Generation Sequencing) in laboratories with access to updated databases. The clinical utility of the screening of other HR genes is still under investigation and more information on their implication is still needed for therapeutic and prevention decisions. Mutations in some of these genes, such as *PALB2, RAD51C*, and *RAD51D* have also been linked to hereditary predisposition to cancer [102]. There is currently no other option for detection of the HR deficiency, not even with immunohistochemistry. The HR signatures look promising, but interpretation of data and the clinical utility is still challenging.

## 2. Description of Mutations in HR Pathways in Breast and Ovarian Cancers

In 2018, the CIMBA (Consortium of Investigators of Modifiers of *BRCA1/2*) consortium presented an inventory of the current state of *BRCA1* and *BRCA2* mutations. There are 1650 unique *BRCA1* and 1731 unique *BRCA2* mutations distributed within these genes [103]. Different types of mutations have been reported: frameshift, nonsense, missense, and splice. Frameshift are the most common type, followed by nonsense mutations. The most common effect of the mutations was premature translation termination and the majority of mutant mRNAs were predicted to undergo nonsense-mediated mRNA decay (NMD) [104]. In the 2000s, large rearrangement (deletion or duplication of one or more exons) were also highlighted [105]. Despite having the same spectrum of mutations, the frequency distribution by mutation type, effect, or function differed significantly (*p* < 0.05) between *BRCA1* and *BRCA2* mutation carriers in the CIMBA cohort [103]. These differences are largely because genomic rearrangements and missense mutations account for a much higher proportion of alterations in *BRCA1* when compared to *BRCA2*, as previously described [106,107].

Mutations in HR genes beyond *BRCA1⁄2* have been reported in breast and ovarian cancers [15,60,61]. As expected, *BRCA1/BRCA2* genes were the most commonly altered genes, followed by several genes, including *CHEK2, PALB2, RAD51C*, and *RAD51D* [108]. Some were preferentially affected by germline alterations (e.g., *BRCA1/2, CHEK2, FANCM, PALB2*), whereas others (e.g., *ATM, BAP1, CDK12*) were preferentially affected by somatic events [109].

The prevalence of germline HR genes alterations in patients with breast cancer is about 10%. After *BRCA1⁄2*, the main HR genes affected are *CHEK2*, *ATM*, *BRIP1*, *PALB2*, *PTEN*, *NBN*, *RAD51C*, *RAD51D*, *MSH6*, and *PMS2* [110]. For ovarian cancer, HR mutations are identified in more than 25% of the cases [95,109]. Beyond *BRCA1⁄2*, the main HR genes affected are *RAD51D, BRIP1, RAD51C, CHEK2, PALB2,* and *BARD1*.

Analysis of TCGA data confirmed the prevalence of HR pathway alterations in 10% and 25% of breast and ovarian cancers, respectively. It was demonstrated that bi-allelic alterations in HR genes are mutually exclusive of each other [87,109]. Analysis of ∼1000 samples confirmed the same pattern of HRD in breast cancer samples of germline (nonsense and frameshift) *PALB2* mutations carriers, while it was not observed in *ATM* or *CHEK2* [109].

### 2.1. Hereditary Breast and Ovarian Cancers

#### 2.1.1. Breast Cancers Related to *BRCA1/2* Mutations

Breast cancers associated with *gBRCA1⁄2* mutations or pathogenic variants represent 3%–5% of cases [111]. The percentage of somatic *BRCA1⁄2 (sBRCA1⁄2)* mutations in breast cancer is not well established. However, two studies found that 3% of unselected cases present *sBRCA1⁄2* mutations [8,96].

About 70% of breast tumors arising in *BRCA1* mutation carriers are “triple negative” [112]. On the other hand, only 10% to 20% of TNBCs carry a *BRCA1* mutation [113,114]. *BRCA1*-associated tumors generally present a higher mitotic rate and are peculiarly higher-grade tumors, presenting greatly increased mitotic count, pushing margins, lymphocytic infiltrate, trabecular growth pattern, and necrosis [115,116,117]. These tumors generally express myoepithelial cell-type cytokeratins (CK5⁄6, CK14, and CK17) and present a basal-like gene expression profile [116]. A previous study showed that reduced expression of CK8⁄18 could help discriminate the basal tumors of *BRCA1* carriers from those sporadic tumors [118].

*BRCA2* breast carcinomas are most closely like sporadic tumors, generally expressing the estrogen receptor (77%) and are in the minority triple negative [112,119]. RNA tumor profiling demonstrated that *BRCA2* tumors are mainly of the luminal B subtype and are more likely than non-*BRCA2* tumors to be ER-positive and of high grade, with pushing margins [120,121].

Mavaddat et al. evaluated the histopathological characteristics of the largest cohort of breast cancer patients harboring *BRCA1/2* germline mutations. This included 4325 patients with *BRCA1* mutations and 2568 patients with *BRCA2* mutations [112]. Breast tumors were mostly invasive ductal carcinomas (occurring in milk ducts) for both *BRCA1* (80%) and *BRCA2* (83%) carriers. Lobular carcinoma (occurring in breast lobules) was the second most common subtype for *BRCA2* carriers (8.4%), and medullary carcinoma (a subtype of invasive ductal carcinoma) for *BRCA1* carriers (9.4%). The frequency of TNBC was 69% for *BRCA1* and 16% for *BRCA2*. HER2-positive was 13% for *BRCA1* and 10% for *BRCA2* tumors. *BRCA1* tumors were a majority grade 3 (77%), while for *BRCA2*, half were grade 3. For *BRCA1* carriers, the grade of the tumor decreased with increasing age, as well as the proportion of estrogen receptor-negative tumors. In contrast, the grade and the proportion of ER-negative tumors increased with age for *BRCA2*. Such findings are in agreement with previous studies with a smaller number of participants [122,123]. Pathological data was available for 702 *BRCA1* and 302 *BRCA2* mutation carriers in the same cohort that developed a contralateral breast cancer [112]. The median interval for a second breast cancer was 5.2 years. Interestingly, ER/PR status of the first breast tumor was predictive of the ER/PR of the second cancer for both *BRCA1* and *BRCA2* carriers, suggesting that the second tumor arises in the same genetic and environmental background with the same pathology.

Concerning the prognosis of *BRCA*-associated breast cancers, recently, the POSH study showed no difference in survival for patients carrying a *BRCA* mutation when compared to those with sporadic breast cancer [124]. However, in the TNBC subgroup, *BRCA* carriers had a better survival than non-carriers, which may be related to better sensitivity to chemotherapy. This survival advantage of the TN *BRCA* mutant subgroup was also confirmed in a recent meta-analysis [125].

#### 2.1.2. Ovarian Cancer Related to BRCA1/2 Mutations

Ovarian epithelial cancers associated with *BRCA1⁄2* mutations represent ~22% of the cases, with 15% germline and ~7% somatic mutations [14]. In the absence of BRCA1 or BRCA2 protein function, the preferential use of error-prone DNA repair mechanisms leads to genomic instability, a peculiar feature of breast and ovarian cancers arising from *BRCA* mutations that may favor carcinogenesis.

The majority of ovarian tumors related to *BRCA1* and *BRCA2* constitutional mutations are serous carcinomas (67%), followed by endometrioid (12%), clear-cell (2%), and mucinous carcinomas (1%) [112]. Tumors in *BRCA1⁄2* carriers are more likely than tumors in age-matched controls to be invasive serous adenocarcinomas and unlikely to be borderline or mucinous tumors. They are of higher grade, with a higher percentage of solid components and are more likely to stain strongly to *TP53* [126]. There are no significant differences in ovarian cancer morphology or grade between *BRCA1* and *BRCA2* tumors [112]. However, *BRCA1* carriers present a higher ovarian cancer lifetime risk than *BRCA2*. The cumulative ovarian cancer risk to age 80 years is around 44% and 17% for *BRCA1* and *BRCA2* carriers, respectively.

Evidence suggests that genomic instability is present in both hereditary and sporadic cancers but occurring in different stages of cancer development and with different molecular basis. While in hereditary cancers, genetic instability probably precedes the acquisition of mutations in oncogenes and tumor suppressor genes, and therefore precedes the acquisition of other hallmarks of cancer [127], studies suggest that the first hallmark acquired in sporadic cancers may be increased activation of growth signaling, secondary to mutations in oncogenes or anti-oncogenes.

According to the mutator hypothesis, genomic instability in hereditary cancers is related to mutations in caretaker genes (genes involved in maintaining genomic stability) that happens during early carcinogenesis. Classical caretaker genes are DNA repair genes, including *BRCA1⁄2*, and mitosis checkpoint genes. Chromosomal abnormalities are present from the stage of precancerous lesions and participate in cancer development by increasing of the spontaneous mutation rate [128]. The observation that only a part of chromosomal abnormalities is seen in all tumor cells is in line with the hypothesis that tumor cells originate from a single genetic unstable cell which continues to accumulate mutations during cancer development. The results of high-throughput sequencing studies showed that mutations in caretaker genes were infrequent in sporadic cancers [129,130,131,132]. However, that inactivation of caretaker genes can also be purely sporadic and define a specific subtype with comparable features of tumors carrying germline mutations. In sporadic cancers, the genetic instability is probably related to an oncogenic-induced collapse of DNA replication forks.

## 3. Unidentified HR Deficiency Mechanisms in Ovarian and Breast Cancers

### 3.1. BRCA1/BRCA2 Variants of Uncertain Significance

Ten percent of individuals undergoing genetic *BRCA1/2* screening receive test results reporting variants of uncertain clinical significance (VUS). These sequence variations are either in-frame deletions/insertions, missense, silent variants, or variants in intronic and regulatory regions that may influence splicing or translation. They present an unknown functional effect on BRCA1 and BRCA2 and cannot currently be classified as either pathogenic or of low clinical significance. A large number of missense variants and virtually all non-coding deep intronic or promoter variants remain of unknown significance (VUS) since they cause subtle changes in protein structure (for missense variants) or in the amount of produced protein (for non-coding variants), being generally difficult to reliably determine their pathogenicity merely from clinical genetic information [133]. A VUS finding should not be considered clinically useful and should not be taken into account for clinical decisions until further evidence emerges to shift interpretation. Medical advice should be solely based on family and personal medical presentation. But in some cases, they are managed inappropriately as pathogenic mutation leading to psychological distress and inappropriate interventions in patients [103]. Even though individual VUS are rare, the identification of a VUS is not a rare event and has a tendency to increase with concomitant sequencing of several genes in NGS panels. Information about VUS is collected in different databases [134,135,136,137]. Attempts to evaluate the clinical significance of these variants include frequency analysis in case-control studies, personal and familial history, co-segregation of the variant with disease in affected families, co-occurrence in trans with deleterious mutations, in silico prediction models, and functional and tumoral data.

*BRCA1/2* VUS classification is particularly challenging. This is why, in 2009, an international consortium was created, which allowed for the classification of a certain number of variants [68,138,139,140,141,142,143,144,145]. This consortium has recently extended the scope of this study for other HBOC genes.

A number of in silico tools are available to help understand if a given intronic or exonic variant leads to an improper exon and intron recognition on messenger RNA and results in the generation of an aberrant transcript of the mutated gene, as well as whether a specific amino acid change may impact protein function [146,147,148,149,150,151,152,153,154,155,156,157]. Functional assays are complementary to in silico prediction, also being used to evaluate the impact of VUS on RNA splicing and/or on protein function [143,153,158,159,160,161,162,163,164,165].

### 3.2. Variants in HR Genes Beyond BRCA1⁄2

Since the discovery of *BRCA1* and *BRCA2* genes 25 years ago, several other breast cancer susceptibility genes have been identified. With the current popularization of next-generation sequencing, the single-gene strategy is used in selected circumstances. Most services are now sequencing *BRCA1⁄2*, along with other genes related to breast and ovarian cancer hereditary predisposition in the context of a HBOC gene panel. These high to moderate penetrance mutations may also contribute to hereditary predisposition, such as *TP53, PTEN, STK11, CDH1, ATM, BRIP1, PALB2*, and *RAD51* isoforms (*RAD51C, D, B*). However, altogether these mutations only explain ~5% of the unsolved cases [108] and VUS are also identified in these new genes. Some of these genes have been found to increase the risk of cancer similar to *BRCA1⁄2*, sharing the same care guidelines for cases where a *BRCA1⁄2* mutation has been identified. *BRCA1⁄2* HBOC can be distinguished from these other disorders based on the spectrum of tumors present in the family and with the aid of germline genetic screening. *PALB2* variants have now been determined to be of high penetrance [15]. *ATM, CHEK2*, and *BARD1* genes are considered to have a moderate increase in risk [166]. Numerous other genes are suspected to be related to the risk of breast cancer, such as *NF1, RAD51C, RAD51D, BRIP1, NBN, MRE11A, FANCM, RECQL, MLH1, MSH2, MSH6,* and *PMS2*. However, they still need confirmation given their low penetrance and divergent results between studies. These previously cited genes are usually included in HBOC panels [102,166]. Still, some of the commercial panels mix genes related to different hereditary cancer syndromes, adding the challenge of interpreting the clinical risk of mutations related to other syndromes when they are identified in HBOC patients. This leads to the increase of uninterpretable results, since the number of variants of uncertain significance increases when multiple genes are tested. For multi-gene panel testing, a mutation is identified in ~30% of HBOC patients, most commonly in *BRCA1* and *BRCA2* [167]. It is thus worth mentioning that despite technology advances and recent democratization of access to genetic screening, the predisposition mechanism remains undefined for about two-thirds of families meeting the clinical criteria for HBOC.

## 4. Management

### 4.1. Risk Reduction Interventions

The importance of identifying at-risk individuals lies in providing appropriate screening, surveillance, and risk reduction interventions. The individualized approach should include discussion about the risks and benefits of risk-reduction surgeries, taking into account patient′s age, priorities, previous cancer history, comorbidities, and cancer-related anxiety. Prospective studies demonstrated that for *BRCA* carriers without a personal history of cancer, bilateral risk-reducing mastectomy (RRM) is associated with 90% or more decreased risk of breast cancer with a residual risk of 1%–2% [168,169]. But the decision to undergo RRM and the ideal time can be influenced by life events, being uncertain for some women. For individuals with HBOC who choose not to undergo risk-reducing surgery, proper follow up with intensive cancer screening has an impact on early detection of cancer with increased cure rate. It is important to mention that for patients with a strong familial breast cancer risk, even if a mutation is not identified, appropriate follow-up and awareness training with monthly self-breast examination should begin at 18 years with clinical breast examination recommended at 25 years, and from then on, every 6 months. Between 25 and 29 years, radiographic screening is suggested. From 30 to 75 years, annual MRI (magnetic resonance imaging) and mammography is recommended. A recent prospective randomized study that performed paired MRI and mammography in women with high risk for breast cancer confirmed the benefit of adding MRI to the screening of this population. This study showed that 61% of the tumors would not have been diagnosed by just a mammography, and it also demonstrated that MRI allowed the diagnosis of cancer at an earlier stage. [170]. For *BRCA1⁄2* mutation carriers older than 50 years, the addition of MRI to mammography improves screening sensitivity by a magnitude similar to that observed in younger women [171].

Ovarian cancer risk should also be taken into account, but screening for ovarian cancer is more challenging due to the low sensitivity of the exams. It is advised that risk-reducing salpingo-oophorectomy (RRSO) be offered between age 35 and 40 years for women with *BRCA1* mutations who have completed childbearing. For *BRCA2* carriers, it can be delayed until the age of 45 years, since only 1% of this population presents ovarian cancer by age 50. Nevertheless, health considerations related to premature surgical menopause, including an increased risk of osteoporosis and cardiovascular disease, should be discussed with women considering surgery. RRSO is the only evidenced-based strategy to prevent ovarian and fallopian tubes cancer. It is associated with an 80% reduction of ovarian cancer risk, a 50% reduction of breast cancer risk in premenopausal women, and of breast and ovarian-cancer specific mortality [172]. Annual screening with CA125 and transvaginal ultrasound may be considered for women who refuse prophylactic surgery, with limited sensitivity (less than 50%) and positive predictive value (less than 17%) [173]. Studies show that both are ineffective in detecting tumors during the very early stage to influence prognosis. The PROSE (Prevention and Observation of Surgical Endpoints) study evaluated the effect of risk-reducing salpingo-oophorectomy on mortality and confirmed that the surgical group had lower all-cause mortality (HR 0.40; 95% CI 0.26–0.61), breast cancer-specific mortality (HR 0.44; 95% CI 0.26–0.76), and ovarian cancer-specific mortality (HR 0.21; 95% CI 0.06–0.80) [168]. Some questions about the extent of surgery remain unanswered, such as whether adding hysterectomy to the procedure has survival benefits, and even if just performing salpingectomy alone would be sufficient for risk reduction. The latter is based on the pathophysiology of ovarian cancer and its likely origin is in situ lesions located in fallopian tubes. Recent data has suggested that women with *BRCA1⁄2* mutations present an increased risk for uterine serous carcinoma, which generated an extensive discussion whether hysterectomy should be performed at the time of prophylactic surgery [174,175]. However, current standard guidelines do not include hysterectomy as part of risk reducing surgery and the decision to perform a concurrent hysterectomy should be individualized [176,177].

Since germline *BRCA2* mutations are associated with a five-to-eight increase in the risk of developing prostate cancers, which are more aggressive and with a shorter survival rate, male carriers should begin prostate screening at age 45. Additionally, *BRCA2* mutations are present in 7% of pancreatic cancers irrespective of familial history, and accounting for ~10% of hereditary pancreatic cancers. Therefore, an individualized screening should be advised, preferably in the context of a clinical screening protocol because there is no consensus for pancreatic cancer screening in most institutions so far.

In addition to intensified screening and risk reduction surgeries, some pharmacological measures have proven to positively impact the management of *BRCA* carriers. Chemoprevention with Tamoxifen may be offered for breast cancer primary prevention of *BRCA2* carriers, since 75% of *BRCA2*-associated breast cancer are ER-positive [178]. However, for *BRCA1* carriers, the current use of tamoxifen is less studied, and data is inadequate to support the use of tamoxifen, since they present mainly TNBC. Beyond Tamoxifen, observational studies have shown that oral contraceptives reduce the risk of ovarian cancer by 30% and 40% in the general and BRCA population, respectively. The concern about theoretical increased risk of breast cancer was not confirmed in studies of women with HBOC syndrome. However, data from randomized controlled trials is lacking and therefore, the use of oral contraceptives for prevention of ovarian cancer in women who have not undergone risk-reducing salpingo-oophorectomy is controversial [179,180].

### 4.2. Implications for Treatment Response: BRCA1/2 Mutations as Predictive Biomarkers

#### 4.2.1. Sensitivity to Platinum Salts

Platinum salts, such as cisplatin and carboplatin, are effective breast and ovarian cancer treatments. They act as DNA cross-linking agents forming intra-strand crosslinks and are especially active in cells lacking HR function. Although their clinical effectiveness as first-line chemotherapy for breast cancers has been confirmed (overall response rate (ORR) of 50% for cisplatin and 30% for carboplatin), studies have shown that they have only modest activity in previously treated metastatic breast cancers [181,182]. Tutt et al. were able to demonstrate that the presence of a germline *BRCA* mutation was predictive of a greater benefit in the metastatic scenario. The trial included 376 unselected TNBC patients after first-line treatment failure who were randomized to receive either carboplatin or docetaxel. While there was no difference between ORR to carboplatin and ORR to Docetaxel in the overall population (ORR 31.4% × 34%), subjects with a *BRCA1⁄2* germline mutation had a significantly better response to carboplatin than to docetaxel, doubling the overall response rate (ORR 68% × 33.3%, *p* = 0.03). However, the highest platinum sensitivity was limited to *BRCA* mutation carriers. Such benefit was neither observed for subjects with a high HRD score, nor for tumors presenting *BRCA1* promoter [183]. These results were consistent with previous results from a smaller phase 2 trial in metastatic TNBC in which platinum agents were active, especially in the presence of *BRCA1⁄2* mutations but not in the presence of *BRCA1* promoter methylation [184].

The activity of platinum salts was also evaluated in early breast cancer, with proven benefits in the neoadjuvant scenario for the TNBC subtype, regardless of *BRCA1* status [185]. Data from a retrospective study support the use of platinum salts in the neoadjuvant treatment of women with a *BRCA* mutation. Expressive response rates have been observed for *BRCA1* mutation carriers treated with cisplatin monotherapy compared with standard regimens based on anthracycline and taxanes (pathological complete response (pCR)= 83% × 8–22%) [186]. However, usefulness is still questionable of *BRCA1⁄2* mutations as predictive biomarkers of platinum response in the neoadjuvant scenario. Some authors advocate that *gBRCA1⁄2* mutation carriers have a higher likelihood of achieving pCR thanks to a higher sensitivity to cytotoxic agents in general, regardless of the addition of platinum salts [187]. GeparSixto was a phase II study which confirmed the benefit of adding carboplatin to neoadjuvant chemotherapy with increase of pathological complete response rate (53.2% × 36.9%, *p* = 0.005), an advantage translated in a superior disease-free survival rate at 3 years. The secondary analysis of GeparSixto trial, performed to evaluate if *BRCA1⁄2* status was predictive of response to chemotherapy, could not confirm this hypothesis. It found that the addition of carboplatin did not increase pCR rate in mutation carriers (65.4% × 66.7% in treated versus untreated, respectively). Surprisingly, in the wild-type population, neoadjuvant carboplatin significantly increased it (55% × 36.4% OR 2.14, 95% CI 1.28–3.58, *p* = 0.004). Additionally, g*BRCA1⁄2* mutation carriers experienced a better disease free survival (DFS), which was not significantly improved by the addition of carboplatin (82.5% in carboplatin treated × 86.3% untreated patients).

Little data is available in the adjuvant setting for platinum salts in *gBRCA1⁄2*-associated breast cancers. In 2014, Dwadasi et al. randomized TNBC patients who had residual disease after neoadjuvant chemotherapy based on anthracycline and taxanes, receiving four additional cycles of adjuvant cisplatin, with and without the PARPi rucaparib [188]. The primary end point was similar in both arms and was not different between patients with BRCA-associated and sporadic tumors (85% × 79%, respectively). Yet it is noteworthy that there was no relapse in any of the eight patients with g*BRCA* mutation.

#### 4.2.2. HR Deficiency and Development of a Targeted Therapy: PARP Inhibitor Treatments

The rationale to use Poly (ADP-ribose) polymerase (PARP) inhibitors to treat tumors harboring *BRCA1⁄2* mutations is based on the principle of synthetic lethality, a concept in which if only one of the two genes is mutated, then it is compatible with viability, while a mutation in both leads to cellular death [189,190].

PARPs are a large family of multifunctional enzymes that play a key role in the repair of single-strand breaks (SSB) through base excision repair. PARP1 is best characterized of the 17 members of the PARP protein family [191]. PARP1 is the major target of PARPi. The inhibition of PARP impairs the repair of SSBs through disruption of the BER pathway and PARP1 trapping that happens through inhibition of auto-PARylation and/or PARP release from DNA. These events lead to accumulation of SSB, which lead to DSBs at the replication fork and thus to the death of homologous recombination-deficient cells such as *BRCA1⁄2* mutants in a process named “synthetic lethality.” This concept has moved from the field of genetics to medical oncology, opening new perspectives for treating tumors containing the BRCAness HR-deficient phenotype.

The first trial evaluating the efficacy of PARPi (olaparib) in breast cancer was published in 2009 [192]. This phase I trial included 60 heavily pretreated women, 3 carrying a *BRCA* mutation. One out of these 3 patients presented a complete response for 60 months. The second had stable disease for 7 months [192]. These results led to the approval of 2 phase II trials, including women with *gBRCA1⁄2* mutations with advanced breast cancer who presented response rates ranging from 12% to 41% [193,194]. Recently, a prospective phase III trial compared olaparib to standard-of-care chemotherapy in patients with metastatic breast cancer harboring a *gBRCA1⁄2* mutation. The PFS was significantly longer in the olaparib group (7 × 4.2 months HR 0.58, 95%CI 0.43–0.80, *p* < 0.001), as well as improvement to quality of life. No significant benefit in overall survival has been proven yet [195].

More recent studies have investigated the benefit of adding platinum salts in comparison and in combination with PARPi for the treatment of BRCA-related early breast cancer. Telli et al. reported a pCR of 36% in a single-arm phase II study that evaluated the combination of iniparib, gemcitabine, and carboplatin for the neoadjuvant treatment of *BRCA* mutation. The study confirmed that a high loss of heterozygosity score was a predictor of better response [196]. Next, the combination of a PARPi (Veliparib) with carboplatin in addition to standard neoadjuvant chemotherapy with Docetaxel was evaluated in the BrighTNess trial, a phase III randomized study that included stage II–III TNBC. In this trial, the addition of carboplatin and veliparib increased pCR rate in both *gBRCA1⁄2* mutation carriers (57%) and wild-type patients (53%), but with no significant differences in patients who received only carboplatin [197].

Just like for early breast cancer, several studies are now comparing platinum salts to PARPi and evaluating them in combination for advanced breast cancer. A recently published phase II trial evaluated the efficacy of adding the PARPi veliparib to chemotherapy regimens (carboplatin and paclitaxel or temozolamide) in patients with *gBRCA1⁄2* mutated metastatic breast cancer. A numerical but not statistically significant increase in progression free survival (PFS) and overall survival (OS) was observed with the addition of veliparib to the platinum-based regimen carboplatin and paclitaxel [198].

Beyond breast cancer, PARPis have been widely tested for ovarian cancer treatment in different settings. High-grade serous carcinoma (HGSOC), the most common subtype of ovarian cancer, is characterized by nearly universal *TP53* mutations (96%) and high genomic instability. As stated before, one half of HGSOC displays defects in the HR DNA repair pathway, with mutations identified in *BRCA1⁄2* in ~22% of the cases with ~15% germline and ~7% of tumoral mutations [14]. Mutations in other HR genes are less common and are present in about 3% of the cases. Sporadic tumors also display HR defects as *BRCA* mutants (the BRCAness phenotype), and consequently higher response rate to platinum-based chemotherapy and PARPi. Most patients with advanced-stage ovarian carcinoma are initially treated with platinum-based chemotherapy, but the majority of them will ultimately relapse. Longer treatment-free intervals and improved overall survival rates observed in this group are related to their inability to repair DNA damage. Based on this rationale, two phase I studies tested the safety and benefit of olaparib for treatment of ovarian cancer harboring *gBRCA1⁄2* mutations [192,199]. In the first study, Fong et al. enrolled 60 solid tumor patients, in which ovarian tumors led with 21 cases. Of the 21 ovarian tumors, all of which received at least one line of chemotherapy, 16 had *gBRCA1⁄2* mutations. Response was only documented in these 16 patients harboring g*BRCA1⁄2* mutations in both platinum-sensitive (61.5%) and platinum-resistant (41.7%) cohorts [199]. This study supported the anti-tumor activity of PARP inhibition for the treatment of ovarian cancer. Subsequently, in the expansion phase, only ovarian cancer carriers of *BRCA1* or *BRCA2* mutations were enrolled. Of the 50 patients, 20 (40%) presented partial or complete response and 3 (6%) presented disease stabilization. The authors further confirmed a significant association between the clinical benefit rate and platinum-free interval.

Subsequent phase II studies confirmed the efficacy of olaparib as monotherapy for the treatment of metastatic HGSOC patients harboring *gBRCA1⁄2* mutations, with ORR ranging from 33%–41% and a median response duration of 8.8 months [200].

Next, the trials focused on the use of olaparib in the maintenance scenario for platinum-sensitive relapsed ovarian cancer. Ledermann et al. confirmed the improvement of PFS by olaparib initially in a retrospective pre-planned analysis of a phase II trial, and subsequently in a prospective trial (8.4 months versus 4.8 months; HR 0.35; 95% CI 0.25–0.49; *p* < 0.001). The benefit was even greater in the presence of *BRCA1⁄2* germline or somatic mutations [201]. Also, SOLO 2 phase III trial met its primary end point, with more improved PFS with olaparib than with placebo (19.1 months versus 5.5 months, HR 0.30; 95% CI 0.22–0.41) [202]. Following these results, olaparib was also tested in newly diagnosed ovarian cancer patients, after administration of platinum-based adjuvant chemotherapy. In the adjuvant scenario, olaparib significantly reduced the risk of disease progression or death by 70% [203].

Therefore, olaparib was initially approved by the Food and Drug Administration (FDA) in 2014 for the maintenance treatment of *BRCA1⁄2*-mutated ovarian cancer. The approval was extended in 2018 to all platinum-sensitive patients, regardless of *BRCA1⁄2* status, because it was realized that the benefit extended to all HRD tumors. Following the SOLO1 trial, olaparib was also approved in first-line maintenance for *BRCA*-mutated (*BRCAm*) advanced ovarian cancer. Currently, two other PARPi, niraparib and rucaparib, have been approved by the FDA for the treatment of ovarian cancer. Other PARPi, such as veliparib and talozaparib, are under development and testing, based on the rationale described above.

The phase III NOVA study confirmed the benefit of niraparib in the maintenance setting of platinum-sensitive HGSOC. The authors stratified the analysis by the presence of *BRCA1⁄2* mutation, and in the wild-type group, by the presence of HR deficiency. The benefit of niraparib was more pronounced among patients with *gBRCA1⁄2* mutation (PFS 21 versus 5.5 months, HR 0.27; 95% CI 0.17–0.41). However, it was not negligible among *gBRCA1⁄2* wild-type patients with HR-deficient tumors (12.9 versus 3.8 months, HR 0.38; 95% CI 0.24–0.59). These results led to FDA approval of niraparib in the maintenance setting, regardless of *BRCA1⁄2* status. Additionally, niraparib antitumor activity was also documented for late-line treatment of ovarian cancer patients, with greater benefit among HRD-positive tumors, regardless of the relation to a *BRCA1⁄2* mutation [204].

Ultimately, rucaparib was also approved by the FDA for maintenance treatment of ovarian cancer, based on the results of ARIEL2 and ARIEL3 trials [89,205]. As for niraparib, a preplanned analysis of PFS, according to a tumor genomic profiling test for homologous recombination and loss of heterozygosis analysis, confirmed that the benefit of the PARPi was bigger but not restricted to *BRCA* mutant tumors. The PFS was 16.6 months and 13.4 months in patients with *BRCAm* and homologous recombination-deficient ovarian carcinomas, respectively (versus 5.4 months for patients who received placebo; *p* < 0.0001).

In line with these findings, it is clear that the population with potential benefit from PARPi is likely wider than germline *BRCA* mutation-associated disease. However, it is known that a portion of the patients even carrying the mutation will present primary or secondary resistance to the treatment. For this reason, biomarkers are in development to broaden the selection of patients, with the potential clinical benefit from these agents.

## 5. Conclusions

A portion of breast and ovarian cancers can be defined by a common pathway involved in the tumorigenesis with an impairment of DNA repair by homologous recombination (HR). However, the reason of the inactivation of this pathway remains unclear for most cases, even in the presence of an HR-deficient signature. It is becoming clear that alternative mechanisms (beyond *BRCA1* and *BRCA2* mutations) can also be used as new biomarkers of therapeutic response. Elucidating them can help improve both genetic counseling and therapeutic response.
